# Community Structure, Species Variation, and Potential Functions of Rhizosphere-Associated Bacteria of Different Winter Wheat (*Triticum aestivum*) Cultivars

**DOI:** 10.3389/fpls.2017.00132

**Published:** 2017-02-13

**Authors:** Aaron K. Mahoney, Chuntao Yin, Scot H. Hulbert

**Affiliations:** ^1^Department of Plant Pathology, Washington State University, PullmanWA, USA; ^2^Molecular Plant Sciences, Washington State University, PullmanWA, USA

**Keywords:** rhizosphere, microbiome, OTU, wheat, microbial community

## Abstract

Minimal tillage management of extensive crops like wheat can provide significant environmental services but can also lead to adverse interactions between soil borne microbes and the host. Little is known about the ability of the wheat cultivar to alter the microbial community from a long-term recruitment standpoint, and whether this recruitment is consistent across field sites. To address this, nine winter wheat cultivars were grown for two consecutive seasons on the same plots on two different farm sites and assessed for their ability to alter the rhizosphere bacterial communities in a minimal tillage system. Using deep amplicon sequencing of the V1–V3 region of the 16S rDNA, a total of 26,604 operational taxonomic units (OTUs) were found across these two sites. A core bacteriome consisting of 962 OTUs were found to exist in 95% of the wheat rhizosphere samples. Differences in the relative abundances for these wheat cultivars were observed. Of these differences, 24 of the OTUs were found to be significantly different by wheat cultivar and these differences occurred at both locations. Several of the cultivar-associated OTUs were found to correspond with strains that may provide beneficial services to the host plant. Network correlations demonstrated significant co-occurrences for different taxa and their respective OTUs, and in some cases, these interactions were determined by the wheat cultivar. Microbial abundances did not play a role in the number of correlations, and the majority of the co-occurrences were shown to be positively associated. Phylogenetic Investigation of Communities by Reconstruction of Unobserved States was used to determine potential functions associated with OTUs by association with rhizosphere members which have sequenced metagenomics data. Potentially beneficial pathways for nitrogen, sulfur, phosphorus, and malate metabolism, as well as antimicrobial compounds, were inferred from this analysis. Differences in these pathways and their associated functions were found to differ by wheat cultivar. In conclusion, our study suggests wheat cultivars are involved in shaping the rhizosphere by differentially altering the bacterial OTUs consistently across different sites, and these altered bacterial communities may provide beneficial services to the host.

## Introduction

The roots of land plants are surrounded by complex communities of microorganisms (Tringe et al., 2005; Peiffer et al., 2013; Edwards et al., 2015). There is a growing body of evidence that the root rhizosphere is a crucial zone for many host–microbe interactions (Zachow et al., 2014; Bulgarelli et al., 2015; Tkacz et al., 2015; Müller et al., 2016; Saleem et al., 2016). Many of these interactions are mutualistic. For example, plants benefit from mobilization of minerals (Vangronsveld et al., 2009; Sessitsch et al., 2013; Plociniczak et al., 2016) and other inorganic molecules, while the microbes benefit from root exudates which provide an energy source in the form of sugars and organic acids (Bais et al., 2006; van Dam and Bouwmeester, 2016). Plant hosts further benefit from microbes with improved growth (Bashan et al., 2014; Glick, 2014), drought and salt tolerance (Castiglioni et al., 2008; Kasim et al., 2013; Han et al., 2014; Naveed et al., 2014; Sarma and Saikia, 2014; Pinedo et al., 2015), and protection against soilborne pathogens (Weller et al., 2002; Cha et al., 2015; De Boer et al., 2015). There is further evidence to suggest that some of these microbial interactions are driven by root exudates, such as malic and hydroxamic acids, benzoxazinoids, and other phytochemicals (Neal et al., 2012; Badri et al., 2013; Carvalhais et al., 2013, 2015; Yin et al., 2013).

The use of high-throughput sequencing and microbial-specific databases have allowed for greater characterization of the bacterial rhizosphere communities. It is possible to classify soil microbes down to the level of species or operational taxonomic unit (OTU) using microbial specific databases and efficient clustering algorithms. Next generation sequencing technologies have allowed for deeper sequencing of the microbial communities over previous methods, such as terminal restriction fragment length polymorphism (TRFLP). These newer approaches have provided better insights into the assemblage of the community in terms of alpha and beta diversity (Peiffer et al., 2013; Edwards et al., 2015). The use of beta diversity analyses, such as ordination, have helped to describe the microbial community patterns over differing habitats such as field locations and by specific host genotypes (Berg and Smalla, 2009; Peiffer et al., 2013; Zachow et al., 2014; Edwards et al., 2015). However, these ordinations only partially address some of the connections between the microbial taxa and their habitats. Newer methods like co-occurrence networks provide better insights into how these species co-occur, either positively or negatively, and the functional roles they have in the habitat (Cardinale et al., 2015).

Recently, co-occurrence networks were used to determine non-random associations between bacterial taxa and their plant hosts (Cardinale et al., 2015; Edwards et al., 2015; Kuebbing et al., 2015; Tu et al., 2016). This method was further complemented with analyzing functional metagenomic data of sequenced bacterial genomes that were associated with species of interest using 16S rDNA markers (Langille et al., 2013; Chen et al., 2016). The informative data these analyses provide could be used to characterize microbial species that may benefit host-plant through environmental services, such as mineral mobilization, nutrient assimilation or antimicrobial production (Wissuwa et al., 2009; Langille et al., 2013; Chen et al., 2016; Raaijmakers and Mazzola, 2016). Microbial studies involving crop species such as maize, rice, and wheat have provided insight into how different bacterial communities respond to host, location, plant growth stage, field management strategies, and soil conditions (Peiffer et al., 2013; Donn et al., 2015; Edwards et al., 2015; Corneo et al., 2016). Studies characterizing the bacterial communities of wheat roots have demonstrated host-dependent and cropping effects. For example, the age of a host plant, and crop rotations can impact the bacterial communities of wheat (Yin et al., 2010; Donn et al., 2015). There is further evidence that tillage management, such as conventional tillage versus minimal tillage may alter the microbial community and their diversity (Navarro-Noya et al., 2013), including the OTU level (Yin et al., 2010). Wheat is grown on more acres than any other crop (FAO, 2015) allowing for an excellent opportunity to manipulate soil biology and microbial communities through host-dependent mechanisms.

We hypothesized that deeper sequencing and more cultivars would provide better insights into host-associated recruitment, and a “bacteria core” for the wheat rhizosphere, particularly in the high rainfall zone of the inland Pacific Northwest (PNW). We also explored the functional potential of the community by using 16S rDNA marker gene associations with species with extensive functional and genomic characterization (Langille et al., 2013; Chen et al., 2016). Lastly, we searched what is known about functions of OTU we found to be responsive to specific wheat cultivars to reveal the potential of wheat breeding to enhance microbial communities for purposes like disease suppression.

## Materials and Methods

### Site Description and Soil Sampling

Two comparable field trials were established on adjacent farms with similar soils but different cropping histories near Pullman, WA, USA. The field sites, approximately 1000 m apart, were established in 2008 (46°47′07.6″N 117°05′21.6″W) at the Cook Agronomy Farm and 2012 (46°46′38.0″N 117°04′57.4″W) at the Plant Pathology Farm. The soil for the two locations is classified as Palouse-Thatuna silt loams, had an average pH of 6.5, and received an average of 462 mm of precipitation annually from 2008 to 2010, and 482 mm from 2012 to 2014 (Supplementary Table 1). The average temperature of the Cook and the Plant Pathology farm for 2008 to 2010 was 8.8°C and for 2012 to 2014 was 9.1°C (Supplementary Table 1). The Cook Agronomy Farm site occupied a slight north facing slope and was previously cropped with spring pea (*Pisum sativum*). While the site at the Plant Pathology Farm was a flat area at the bottom of mild north and south facing slope, and was previously cropped with a mix of winter wheat cultivars. Plot locations were fertilized using a commercial formula (McGregor Company, Colfax, WA, USA) mixed at an 8–3 ratio. This mixture was applied at a rate of 89.6 kg of nitrogen, 16.8 kg of phosphorus, and 13.5 kg of sulfur per hectare. Winter wheat lines varying in pedigree (**Table 1**), were grown in 1.5-m × 1.5-m plots. Four of the lines consisted of two pairs of near isogenic lines either carrying the *ALMT1* (ALuminum-activated Malate Transporter 1) gene in the Chisholm or Century background (PI561722 and PI561725, respectively) or the *almt1* allele in the same backgrounds (PI561726 and PI561727, respectively) (Sasaki et al., 2004; Lakshmanan et al., 2013). An additional five lines were well-established wheat cultivars in the PNW, grown on many acres, and determined in greenhouse tests to have different effects on suppression of *Rhizoctonia* species, possibly through alteration of microbial communities (Mazzola and Gu, 2002; Gu and Mazzola, 2003). Approximately 250 g of each cultivar (1000-seed weights, **Table 1**) were hand sown into five manually dug, 5 cm deep trenches during October of each year. Plots of each cultivar were replicated three times in a randomized block design for both sites. In the first growing season, whole plants were harvested at maturity approximately 10-cm from the ground during September of each year and the same cultivar replanted into previous plant stubble as to best mimic a continuous minimal tillage system. Plots were managed by manually removing weeds.

**Table 1:** Pedigrees of winter wheat cultivars that were tested for associated operational taxonomic units (OTUs) at two locations and 1000-seed weights for each cultivar.

Cultivar	Pedigree	Market Class^1^	Year^2^	1000-seed weight^3^
Eltan	Luke//BR-70443-4, PI167822/CI013438	SWW	1990	41.1
Finch	Dusty/IWA7164/Dusty	SWW	1987	40.5
Hill81	YAMHILL/HYSLOP	SWW	1981	41.2
Lewjain	LUKE(CI-14586)/WA-5829	SWW	1982	37.6
Madsen	VPM-1/MOISSON-951//2^∗^HILL-81	SWW	1988	40.1
PI561722	Chisholm^∗^4/Atlas 66 (Al tolerant)	HRW	1992	41.5
PI561725	Century^∗^4/Atlas 66 (Al tolerant)	HRW	1992	40.6
PI561726	Chisholm^∗^4/Atlas 66 (Al susceptible)	HRW	1992	39.8
PI561727	Century^∗^4/Atlas 66 (Al susceptible)	HRW	1992	40.2

*^1^Market class abbreviation; hard red winter (HRW) and soft white winter (SWW). ^2^Refers to the year that cultivar or germplasm was released. ^3^Mass of 1000 seeds in grams.*

### Soil Rhizosphere Collection

In the 2nd year for each trial, roots from five to ten randomly selected individuals were harvested at the reproductive stage from each plot in late May to early June. Samples were collected by manually digging around each plant to a depth of 20–30 cm. Tillers and bulk plant material were cut and roots with a partially attached stem were then placed into plastic bags. For 2010 and 2014, root samples were collected from five plants of each cultivar per plot. Roots with the majority of the bulk soil removed were placed into a 50-ml plastic tube with 20 ml of sterile distilled water. To collect the rhizosphere soil, each tube was vortexed for 60 s and sonicated for an additional 60 s. Bulk roots were then removed from the tubes using sterile forceps and the tubes centrifuged at 10,000 *g* for 5 min to pelletize the soil. After the supernatant was removed, the pellet (approximately ≤ 0.25 g) was added to a PowerBead tube, a component of the PowerSoil DNA isolation kit (Mo Bio, Carlsbad, CA, USA). DNA was extracted following manufacturer protocol. DNA was stored at -20°C.

### Sequencing and Processing of Reads

DNA samples were sent to Molecular Research (MRDNA, Shallowater, TX, USA). The V1–V3 region of the 16S rDNA gene was amplified using barcoded 27F (5′-AGAGTTT GATCMTGGCTCAG-3′) and 518R reverse (5′-ATTACCGCG GCTGCTGG-3′) primers. These primers were chosen as the V1–V3 region has been to found to provide good coverage for bacterial community reconstruction (Yu and Morrison, 2004; Kumar et al., 2011), and captures high levels of rhizosphere soil diversity for bacterial taxa (Peiffer et al., 2013). Amplification was performed using HotStar Taq Plus Master Mix Kit and the following conditions: 94°C for 3 min; following 28 cycles of 94°C for 30 s, 53°C for 40 s, and 72°C for 60 s, with a final elongation step at 72°C for 5 min. Samples were purified and pooled after being checked on a 2% agarose gel for relative intensity and size.

Libraries were prepared using the Illumina TruSeq DNA library kit and following manufacture’s protocol. Amplicons were paired-end sequenced using 300 bp chemistry on the Illumina MiSeq, using standard sequencing controls. Sequences were processed using the Molecular Research’s pipeline. Briefly, paired-end reads were merged, denoised and samples with a quality score < 25 and homopolymers > 8 were removed. Samples were de-multiplexed and downloaded for use with sequence reads separated into samples (sequences per sample, Supplementary Table 2).

### Operational Taxonomic Unit Processing and Assignment

Sequences from each sample were trimmed of primers, barcodes, and to a length of 350 nt from the 3′ end. Sequences less than 350 nt were removed. Samples were merged into a single file for OTU clustering. Sequences were subjected to chimeric detection using UCHIME implemented in USEARCH ver. 7 (Edgar, 2013). Filtered sequences were then trimmed and clustered into OTUs using a >97% similarity and the UPARSE pipeline (Edgar, 2013) with the removal of singletons.

The Ribosomal Database Project (RDP) classifier (Cole et al., 2009) was used to classify the most abundant representative OTU sequence with RDP (ver. 2.12) and Greengenes 16S rDNA references using MICrobial Community Analysis (MICCA, Albanese et al., 2015). Sequences that classified as plastidal (e.g., chloroplast) or mitochondrial were removed from further analysis. The OTU table was normalized by two methods; samples were rarefied to a read depth of 20,365 and relative abundance was calculated by dividing the number of each OTU by the total number of sequences in a sample. To identify OTUs that occurred in 95% of the wheat rhizosphere samples, QIIME (Caporaso et al., 2010) and the script, compute_core_microbiome.py was used.

### Co-occurrence Networks and Functional Metagenome Predictions

The co-occurrence networks were constructed using Cytoscape (Shannon et al., 2003) and the CoNet plug-in (Faust et al., 2012). Spearman’s correlations using thresholds set at ≥ 0.8 or ≤-0.8, and a *P* < 0.01 were used to determine correlations.

The functional metagenome predictions were based on the algorithm implemented in Phylogenetic Investigation of Communities by Reconstruction of Unobserved States (PICRUSt) 1.0.0 (Langille et al., 2013). The sequences were clustered and assigned to the Greengenes reference (ver. 13.5) at a 97% similarity level. The rarefied OTU table and the PICRUSt standard operating protocol with scripts, normalize_by_copy_number, predict_metagenomes, and categorize_by_function, were used to produce a table of the functional gene content of KEGG Orthology (KO) terms and pathways, defined to level 3. Sulfur, phosphate, nitrogen, and antimicrobial KEGG pathways were chosen to determine genotypic enrichment. Sequence counts associated with malate related pathways were analyzed to determine if there were differences in bacterial responses by the aluminum tolerant wheat cultivars. Reference pathways were searched in the KEGG database^4^ and all associated KO terms for specific pathways were compiled and used for wheat cultivar analysis.

### Statistical Analysis

To determine significant differences in the relative abundances of cultivar-associated phyla, OTU, locations and their interactions, data was log(x+1) transformed, and a permutational multivariate analysis of variance (PERMANOVA) test in PRIMER (v7, PRIMER-E, Plymouth, UK) was run, using a Bray–Curtis similarity resemblance and 999 permutations. Any significant differences were further tested using pair-wise tests in PRIMER. All other statistical differences between samples were compared using paired *t*-tests, analysis of variance (ANOVA), or a non-parametric test (Kruskal–Wallis test) in JMP (ver 12. SAS, NC). Tukey’s honestly significant difference (HSD) or Steel-Dwass All Pairs implemented in JMP12 were used to determine cultivar differences based upon parametric or non-parametric one-factor data, respectively. An OTU relative abundance cut-off of 0.01% was used to calculate wheat cultivar differences and a Benjamini–Hochberg false discovery rate (FDR) at *P* < 0.10 [corresponding to an uncorrected alpha (*P*) = 0.02] was used to correct for multiple hypothesis testing. To determine the difference for wheat cultivars based on the *post hoc* analysis, a wheat cultivar with the same letter and no significant difference across locations, were indicators for an effect.

Principle Coordinate Analysis (PCO) was conducted using OTU relative abundances in the rhizosphere of different wheat cultivars and the two field sites. The PCO was visualized by using log(x+1) data transformation and Bray–Curtis resemblances measures in PRIMER. OTUs with significantly different frequencies across cultivars or locations were plotted using Pheatmap in R. Spearman correlations for network analysis were conducted by plotting rank-order of relative abundance against the number of co-occurrences in JMP12. Rarefied sequence count data (required for PICRUSt) was used for metagenomics functional analyses. KO terms for different wheat cultivars were compared using data from PICRUSt and analyzed in the program Statistical Analysis of Taxonomic and Functional Profiles (STAMP, Parks and Beiko, 2010). The Benjamini–Hochberg FDR was used to correct for multiple testing. Differences were further tested in JMP (ver.12) using Tukey’s HSD to determine wheat cultivar effects.

## Results

### Rhizosphere OTU Composition and Core Bacteria

**FIGURE 1:**
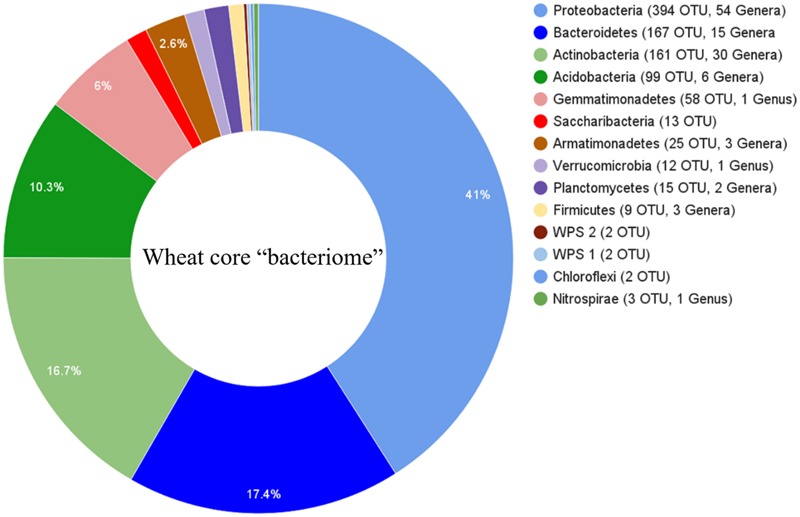
**Phylogenetic distributions of bacterial operational taxonomic unit (OTUs) that were presented in at least 95% of rhizosphere samples across all cultivars and their locations.** Phyla are listed with number of OTUs and the number of classifiable taxa associated with each phylum.

Sequence comparisons of the V1–V3 region of 16S rDNA genes were used to assess the bacterial rhizosphere communities of nine wheat cultivars grown in two locations. After trimming and quality filtering, a total of 5,522,528 reads 350 bp in length were obtained from the 54 rhizosphere samples. These sequences clustered into 26,604 OTUs using a 3% dissimilatory threshold. The core rhizosphere consisted of 962 OTUs, which were found in 95% of wheat cultivar samples, and included phyla Proteobacteria (with 394 OTUs), Bacteroidetes (167), Actinobacteria (161), Acidobacteria (99), Gemmatimonadetes (58), Armatimonadetes (25), Planctomycetes (15), Candidatus Saccharibacteria (13), Verrucomicrobia (12), Firmicutes (9), Incertae_sedis (4), Nitrospirae (3) and Chloroflexi (2) (**Figure 1**).

### Comparative Analysis of Wheat Cultivars

**FIGURE 2:**
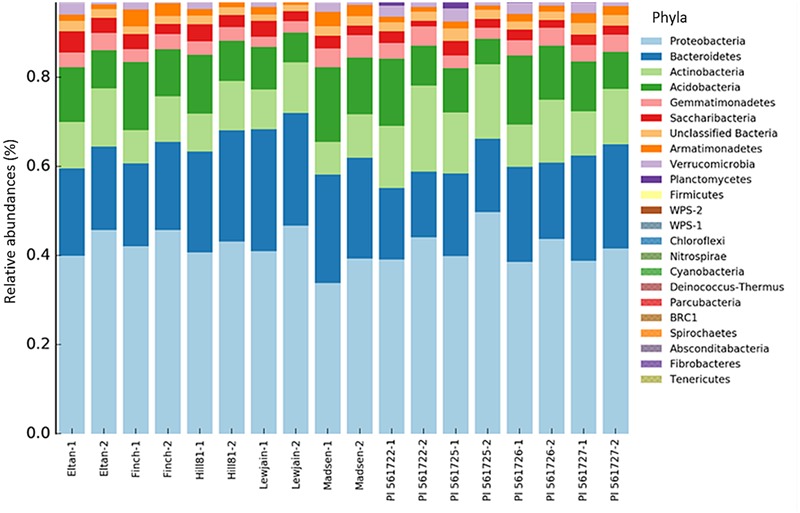
**Bacterial taxonomic assignments at the phyla level and their percentage contribution (abundance) in the rhizosphere soil of different wheat cultivars at two locations (Cook Farm, 1, and Plant Pathology Farm, 2)**.

Across the nine wheat cultivars, 26 Phyla were found using the RDP classification. Of these phyla, two comprising more than 65% of the total sequence reads were identified as Proteobacteria (mean = 49.4%) and Bacteroidetes (mean = 20.8%). Differences in the relative abundance for certain phyla were found between the wheat cultivars. A higher abundance for *Planctomycetes* (*P* < 0.001) was observed for Eltan, PI561722, and PI561725. Cultivars Finch, Madsen, and PI561726 had more classified *Acidobacteria* relative abundances (*P* = 0.001) in their rhizospheres. The phyla *Actinobacteria* was found to be in greater abundance in the rhizospheres of PI561722 and PI561725 (*P* = 0.001). Smaller differences in abundance were observed for C*hloroflexi* (*P* = 0.01), *Fibrobacteres* (*P* = 0.021), and *Verrucomicrobia* (*P* = 0.041) for multiple wheat cultivars (**Figure 2**).

**FIGURE 3:**
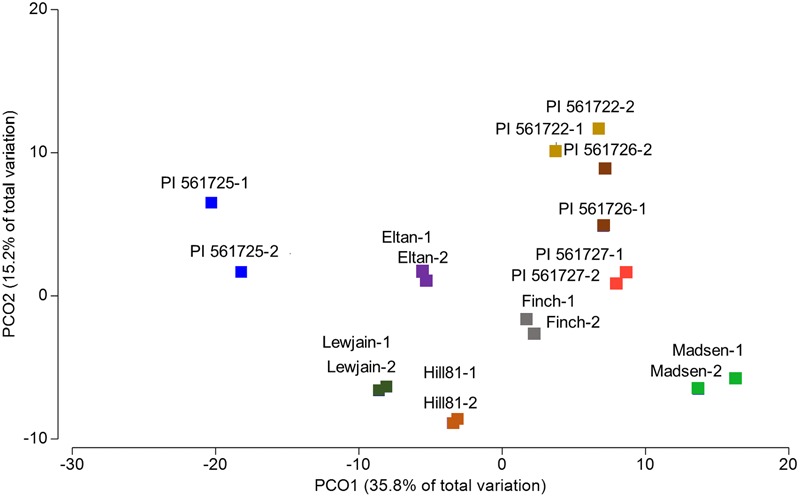
**Principal coordinate analysis (PCO) based on rhizosphere abundances of OTUs for cultivars which were sampled in two locations (Cook Farm, 1, and Plant Pathology Farm, 2)**.

To investigate if there were significant differences in the relative abundances of cultivar- associated OTUs, a PERMANOVA test statistic was conducted to determine the effect of plant cultivar and field site on variation in the relative abundances of the OTUs. A significant effect was found for cultivar (*P* = 0.001, 997 perms,) and for location (*P* = 0.007, 999 perms). No significant interaction was determined by these two factors (*P* = 0.875, 997 perms, **Table 2**). To visualize these differences, a principle coordinate analysis plot was produced using relative abundances from the Bray–Curtis similarity measures. Several of the wheat cultivars were well separated indicating differences in the relative abundances of OTUs in their rhizosphere communities (**Figure 3**). The first two principle coordinates explained about 51% of the variation among the 18 cultivar/location treatments. Axis one explained the major source of variation (35.8%) and appeared mainly to correspond to differences in host cultivar (**Figure 3**).

**Table 2:** Permutational multivariate analysis of variance (PERMANOVA) of main factors tested and their interactions for the wheat rhizosphere.

Sources of variation		Df	Sum of squares	Mean squared	Pseudo-*F*	*P-*value	Permutations
Phyla							
	Cultivar	8	1808	106.4	1.69	0.013	990
	Location	1	1408	151.2	1.12	0.252	999
OTU^a^							
	Cultivar	8	12840	1604.9	2.23	0.001	997
	Location	1	2502.1	2502.1	3.47	0.007	999
	Cultivar × location	8	1049.9	131.2	0.18	0.875	997

*^a^Operational taxonomic unit (≥ 97% sequence similarity).*

**FIGURE 4:**
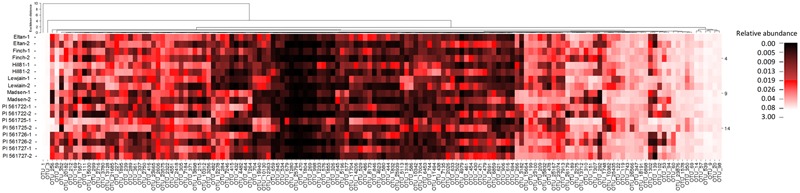
**A heat map displaying the frequencies of OTUs with an average relative abundance ≥ 0.01% in rhizosphere soils of different cultivars listed are noted by the two different locations (Cook Farm, 1, and Plant Pathology Farm, 2) in which they were sampled**.

To identify host-associated OTUs that were different between the nine cultivars, and provide a more reproducible analysis, we removed from the analysis all OTUs which did not have a relative abundance greater than 0.01% when averaged over all treatments. This reduced the number of OTUs from 26,604 to 1305. Of these 1305, differences with location and/or wheat cultivar were found for 148 OTUs (*P* < 0.01, **Figure 4**). Twenty-four OTUs, representing 2.4% of the total normalized rhizosphere sequences, were found to be significantly enriched or depleted on specific wheat cultivar in both fields (**Table 3**). The cultivar-associated OTUs were not limited to a specific clade but rather came from most of the abundant phyla, including *Acidobacteria* (2 OTU), *Actinobacteria* (2), *Bacterioidetes* (9), *Gemmatimonadetes* (3), *Proteobacteria* (8), and *Verrucomicrobia* (1) (**Table 3**).

**Table 3:** Twenty-four cultivar-associated OTUs using a false discovery rate (Benjamini–Hochberg) and Tukey’s HSD for wheat cultivars grown at two farms for two consecutive years.

OTU_ID^1^	Phylum	Class	Genus	Accession^2^	Cultivar-associated^3^
OTU_212	Acidobacteria	Acidobacteria Gp1	Gp1	JX694540	Hill81
OTU_17004	Acidobacteria	Acidobacteria Gp1	Gp1	JQ381822	Hill81
OTU_59	Actinobacteria	Actinobacteria	Amycolatopsis	DQ076483	Hill81
OTU_10130	Actinobacteria	Actinobacteria	Arthrobacter	KM434258	PI561725
OTU_1302	Bacteroidetes	Flavobacteria	Chryseobacterium	JX987481	PI561725
OTU_256	Bacteroidetes	Flavobacteria	Flavobacterium	NR133746	PI561725
OTU_292	Bacteroidetes	Sphingobacteria	Dyadobacter	EF516729	PI561725
OTU_310	Bacteroidetes	Sphingobacteria	Ferruginibacter	JF139728	PI561725
OTU_19940	Bacteroidetes	Sphingobacteria	Ferruginibacter	JX172038	PI561725
OTU_1318	Bacteroidetes	Sphingobacteria	Mucilaginibacter	AF409002	Lewjain
OTU_1748	Bacteroidetes	Sphingobacteria	Mucilaginibacter	HE819800	Madsen
OTU_11521	Bacteroidetes	Sphingobacteria	Pedobacter	HM049699	PI561725
OTU_743	Gemmatimonadetes	Gemmatimonadetes	Gemmatimonas	HQ118407	Madsen
OTU_347	Gemmatimonadetes	Gemmatimonadetes	Gemmatimonas	JQ369813	Madsen
OTU_15664	Gemmatimonadetes	Gemmatimonadetes	Gemmatimonas	HQ118407	Madsen
OTU_119	Proteobacteria	Alphaproteobacteria	Methylosinus	JF910921	Madsen
OTU_1895	Proteobacteria	Alphaproteobacteria	Microvirga	JX777929	Hill81
OTU_1832	Proteobacteria	Alphaproteobacteria	Sphingomonas	JX776177	PI561725
OTU_6898	Proteobacteria	Alphaproteobacteria	Sphingomonas	AM936331	PI561727
OTU_252	Proteobacteria	Betaproteobacteria	Shinella	AM697120	PI561725
OTU_11409	Proteobacteria	Betaproteobacteria	Variovorax	KM877167	PI561725
OTU_744	Proteobacteria	Gammaproteobacteria	Dokdonella	JF910515	Hill81
OTU_13128	Proteobacteria	Gammaproteobacteria	Lysobacter	JX112990	Lewjain
OTU_1134	Verrucomicrobia	Opitutae	Opitutus	HM723529	Madsen

*^1^Operational taxonomic unit (OTU) were assembled based on 97% sequence similarity.*

*^2^Closest BLASTn match to the NCBI database using the most abundant OTU representative and selecting the first or lowest *E*-value.*

*^3^The cultivar in which the OTU was most highly associated (abundant).*

### Observations with Cultivar-Associated OTU and *Rhizoctonia* Suppression

To identify cultivar-associated OTUs in our dataset which correspond to bacterial isolates shown to be suppressive to the soilborne pathogen *Rhizoctonia solani* AG8, the most abundant V1–V3 sequence from the cultivar-associated OTUs were pairwise aligned with the cloned sequences from the suppression study. The previously isolated bacteria came from a very different soil type from a much lower rainfall region of Washington. OTU_1302 (**Table 3**) had a 97.2% sequence identity to the isolate 31 of *Chryseobacterium soldanellicola* (previously designated OTU437) indicating isolate 31 is a representative of this OTU. A second isolate of *C*. *soldanellicola* that was found to be suppressive (previously designated OTU18, isolate 43), had the closest sequence to OTU_1302 but was less than 97% identical (96.2%). OTU_11521 (**Table 3**) had a 91.7% sequence similarity with the isolated strain of *Pedobacter* sp. (previously designated OTU26). The *Pedobacter* isolate (OTU26) was found to belong to the current OTU_8489 (99.13% sequence identity). This OTU was found to have a relative abundance of 0.26% but was not found to have a significant cultivar or location effect. One other isolate, a suppressive *Pseudomonas* OTU (previously designated OTU9) was found to share sequence similarity with OTU_13 at a 97.8% identity. OTU_13 was not found to be cultivar-associated, but had an average relative abundance of 0.73%.

### Co-occurrence Networks Analysis of Rhizosphere

**FIGURE 5:**
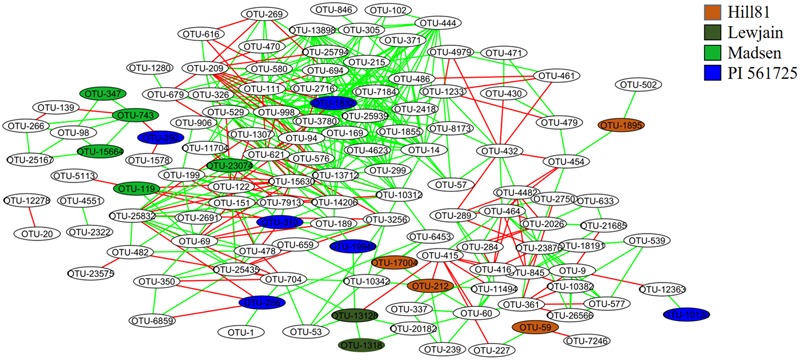
**Co-occurrence networks of the rhizosphere associated bacterial community from different cultivars sampled at two locations.** Nodes are labeled by OTU number and are colored by host associated cultivar. The green and red lines specify significant positive and negative correlations (Spearman correlations ≥ 0.8 or ≤-0.8, *P* < 0.01) between two nodes, respectively.

Co-occurrence interactions between bacterial taxa were examined to shed light on which taxa responded at the genera and OTU level to provide a means toward examining the environmental traits causing the variation. When the OTUs were grouped into their respective genera, 204 significant correlations (edges) were identified between 95 nodes (genera). There were strong co-occurrence patterns between members of classes *Alphaproteobacteria* (genera *Methylovirgula* and *Acidiphilium*), *Betaproteobacteria* (genus *Collimonas*), *Gammaproteobacteria* (genus *Serratia*), *Actinobacteria* (genus *Frankia*), and *Sphingobacteria* (genus *Mucilaginibacter*). The classes *Actinobacteria* and *Sphingobacteria* were found to be most abundant and most interconnected, respectively (Supplementary Figure 1). Since not all members of a genus respond similarly, a co-occurrence analysis was performed on individual OTUs using the 148 that showed variation between treatments (cultivar or location). When OTUs that differed by treatment were examined for co-occurrence, 127 OTUs (nodes) out of 148 were involved in 505 co-occurrences (**Figure 5**). The four OTUs with the greatest positive connectivity were OTU_3780 (26 links, 24 positive), OTU_13898 (25 links, 24 positive), OTU_1832 (24 links, 22 positive), and OTU_998 (24 links, 22 positive). The OTUs 3780, 13898 and 1832 belonged to the genus *Sphingamonas* and OTU_998 belonged to *Segetibacter*. OTU_621 and OTU_209, had 14 (21 links) and 12 (16 links) negative correlations and belonged to genera *Burkholderia* and *Rhodoblastus*, respectively. OTUs 347, 743 and 15664, all belonging to the genus *Gemmatimonas*, were shown to co-occur and be most closely associated with cultivar Madsen. A similar association was observed for OTU_13128 and OTU_1318, belonging to genera *Mucilaginibacter* and *Lysobacter*, which were most closely associated with Lewjain. In contrast, two OTUs 743 (*Gemmatimonas*) and 292 (*Dyadobacter*) were shown to co-exclude and were most closely associated with Madsen and PI561725, respectively. This suggested the host may have factored into their co-exclusion. No correlations were found for seven (OTUs 744, 1134, 1302, 6898, 1748, 11521, 11409) out of the 24 cultivar-associated OTUs in the network analysis (**Figure 5**). This suggested these OTUs responded more uniquely to the cultivar and location treatments. Relative abundance did not serve a role in the number of co-occurrences and their connectivity (Spearman *R* = 0.228, *P = 0.453*).

### Predicted Gene Functions for Bacterial Communities by Wheat Cultivars

To characterize members of the rhizosphere community with sequenced metagenomics data, PICRUSt was used to infer 16S rDNA rhizosphere sequences to available sequenced bacterial genomes. These metagenomic interpretations were categorized into known functions associated with sequenced communities using the KEGG database and sequence counts were separated for each wheat cultivar. From the initial analysis, 2552 KEGG orthology (KO) terms were found to be significantly different for wheat cultivars.

Annotations classified into 329 functional KO pathways (level 3). Many of these pathways were not plant associated, so we chose to focus on KEGG pathways or KO functions related to bacterial metabolism that could benefit the plant host, and test the role of the malate root exudates. The pathways chosen were associated with sulfur, nitrogen, malate, phosphorus, and antimicrobial production. From this analysis, 48 KO terms were found to differ for the microbial sequence counts by wheat cultivar. A *post hoc* analysis demonstrated 12 of these terms were found to have a cultivar effect for both locations (*P* = 0.02). KO terms for the production of antibiotics streptomycin, tetracycline, and beta-lactam were significantly different for sequence counts by wheat cultivar (**Table 4**). Wheat cultivar Madsen was found to have the highest number of sequence counts for the antibiotic KO terms, with genera *Mucilaginibacter* and *Pseudomonas* contributing 26% of the phenotypic variation. A total of six sulfur associated metabolism terms were found to be significantly different by wheat cultivar. Three terms in the sulfur metabolism pathway, sulfate reductase, sulfate adenyltransferase subunits 1 and 2 were found to have the greatest number of sequence counts for cultivar Madsen. In contrast, PI561725 was found to have the lowest number of sequence counts for these three terms. The genera *Mucilaginibacter, Pseudomonas*, and *Gemmatimonas* contributed 33% of the total variation. The wheat cultivars Lewjain and Madsen were found to have the highest numbers of sequence counts associated with nitrogen metabolism. The genera *Prevotella* and *Fusobacterium* contributed 41% of the phenotypic variation. Madsen and PI561725 were found to have the highest sequence counts for phosphate metabolism terms inositol oxygenase and inosose dehydrogenase, respectively. The highest number of sequence counts for the malate dehydrogenase enzyme belonged to cultivars PI561722 (*N* = 72) and PI561725 (*N* = 255), the two members of the isoline pairs that carried the *ALMT1* allele (*P* = 0.004, **Table 4**).

**Table 4:** Phylogenetic Investigation of Communities by Reconstruction of Unobserved States (PICRUSt) 16S rDNA of rarefied functional counts for cultivar associated KEGG KO terms for the wheat rhizosphere soils grown at two farms for two consecutive years.

		Wheat cultivars									
Kegg Pathway	Kegg orthology term^a^	Eltan	Finch	Hill-81	Lewjain	Madsen	PI561722	PI561725	PI561726	PI516727	*P-*value
Antimicrobial	Beta lactam production	41231	44570	42661	39127	50088	44528	35857	45102	41163	0.013
	Tetracycline production	95736	99922	97660	93830	106610	96432	88107	101324	97863	0.019
	Streptomycin production	43607	45411	43408	41638	48939	47223	40034	49039	44627	0.021
Sulfur	Cystathionine synthase	13343	13341	13396	12552	15087	15589	12282	15497	13258	0.002
Metabolism	Sulfate reductase	17152	19529	17900	17050	21289	17348	14042	20219	17981	0.016
	Sulfate adenylyltransferase 1	13162	13743	13244	12242	15072	13709	11397	14241	12945	0.006
	Sulfate adenylyltransferase 2	20739	21001	20460	19981	22507	21888	19490	21668	21137	0.005
Nitrogen	Nitric oxide reductase	4139	4112	4143	4783	4349	3652	4162	3736	4301	0.007
Metabolism	Glutamate synthase	38235	39698	39147	37301	43327	41757	36778	42049	40366	0.002
Phosphate	Inositol oxygenase	3746	4971	4354	4089	5673	3775	2476	5494	4724	0.001
Metabolism	Inosose dehydratase	9264	8500	8353	9518	7918	10995	11270	9417	9519	0.002
Malate	Malate dehydrogenase	62	22	27	48	19	78	255	42	58	0.004
Metabolism											

*^a^For each orthology term, the counts are an average of the two sites (three replications per wheat cultivar per site). If there were statistical differences for a cultivar by location, these KO terms were removed from further analysis.*

## Discussion

The reduction of environmental and financial costs associated with conventional agriculture requires novel management and breeding strategies that aim to shift current high input methods to more sustainable biological methods. Breeding for wheat cultivars that can recruit beneficial microbes could potentially reduce inputs and make production more sustainable. In this work, we characterized the rhizosphere bacteria associated with nine field-grown winter wheat cultivars by comparing the V1–V3 region of the 16S rDNA gene. This method of sampling and analysis allowed for testing host cultivar effects on the rhizosphere bacteria. OTU variation between wheat cultivars was characterized, and 24 out of the1305 most abundant OTU were found to vary in frequency in the rhizospheres of the different wheat cultivars. This result indicated that the host genotype played a minor but significant role in the bacterial diversification of the rhizosphere and that bacterial frequencies can be altered by selecting specific wheat cultivars.

In previous reports, wheat cultivars Lewjain, Eltan, and Hill81 have been shown to differentially select members of the *Pseudomonas* spp. (Mazzola and Gu, 2002: Gu and Mazzola, 2003; Okubara et al., 2004). Interestingly, no significant differences for *Pseudomonas* spp. were observable for the same wheat cultivars in the present study. However, members of the *Pseudomonas* were present in the rhizosphere, accounting for 0.7% of the representative OTUs, and 11 OTUs in the core bacteriome (**Figure 1**). Mazzola and Gu (2002) demonstrated Lewjain had a tendency to make soils more suppressive to soilborne pathogens like *Rhizoctonia* spp. and in soils conducive to apple replant disease (Gu and Mazzola, 2003), as compared to other wheat cultivars like Eltan and Madsen. They provided evidence this suppression was mitigated through host-associated recruitment of *Pseudomonas* spp. into the rhizosphere. Biocontrol studies have often looked at using species like *Pseudomonas fluorescence* for their production of antimicrobial compound 2,4-diacetylphoroglucinol (2,4-DAPG) (Landa et al., 2003). Interestingly, Landa et al. (2003), observed species of genera *Arthrobacter, Chryseobacterium*, and *Flavobacterium* to be enriched in the presence of 2,4-DAPG producing species, such as *P. fluorescence*. In the present study, we identified OTU_10130 (genus, *Arthrobacter*), OTU_1302 (*Chryseobacterium*), and OTU_256 (*Flavobacterium*) to be enriched in the rhizosphere of some wheat lines, especially line PI561725. Although, in significantly lower abundance than PI561725, OTU_1302 was observed to be in greater abundance for the wheat cultivar Lewjain than for other cultivars. However, we did not see the same higher relative abundance for OTUs 256 and 10130. It is likely that the different soil types and their microbial communities, or the methods employed in the previous studies, such as using a greenhouse or growth chamber, may have attributed to differences in these studies.

Differences in the rhizosphere communities of various crop species have been observed (Aira et al., 2010; Bouffaud et al., 2012; Peiffer et al., 2013; Edwards et al., 2015), including differences with two wheat cultivars using TRFLP on the V1–V3 region (Donn et al., 2015). Interestingly, our results differed from a recent report by Corneo et al. (2016) on 24 wheat lines which found no microbial community differences at the vegetative stages of wheat using the TRFLP method on amplicons generated from the V3–V6 hypervariable regions. The lower resolution by the TRFLP method may have affected their ability to identify community differences between wheat lines. In this study, as well as the work by Donn et al. (2015), rhizosphere samples were collected at the reproductive stages and examined after 2 years of growth in the same soil. In previous studies, no differences were found in the first planting cycle (Donn et al., 2015), or in the vegetative stages (Donn et al., 2015; Corneo et al., 2016) of wheat. Several lines of evidence suggest bacteria are recruited through root exudates, and the greatest release may occur during the reproductive stages (Steinkellner et al., 2007; Rudrappa et al., 2008b; Ferluga and Venturi, 2009; Zhang et al., 2009; Neal et al., 2012; Lakshmanan et al., 2013). Multiple growth cycles of different wheat genotypes to recruit responsive microbes may be necessary to see noticeable changes in their frequencies, depending on their initial frequencies in the soil. Changes of frequencies in bulk soil, not just soil closely associated with wheat roots, would presumably take longer but wheat genotypes with stronger effects on microbial communities may be able to affect these communities more rapidly.

Plants have evolved mechanisms to tolerate the toxic effects of aluminum by excreting organic acids into rhizosphere soils (Kochian et al., 2004). In wheat, the gene *TaALMT1*, an Al-activated malate transporter has been found to confer such a tolerance (Sasaki et al., 2004). A previous study involving an aluminum tolerant (Atlas 66) and intolerant line (Scout 66) found no significant differences after 60 days for the rhizosphere communities using DGGE methods (Wang et al., 2013). We compared two sets of isolines and found very noticeable differences in the Century background (PI561725 and PI561727) but not in the Chisholm background (PI561722 and PI561726; **Figure 3**; **Table 3**). Differences in the genetic background of these pairs could have attributed to the observable changes through a differential response to aluminum or an induced root defense response (Rudrappa et al., 2008a; Millet et al., 2010). Lakshmanan et al. (2012) reported the bacterial MAMP flg22 or the phytotoxin coronatine can induce malic acid expression in *Arabidopsis thaliana* in the absence of a low pH or an aluminum rich environment. Additional experiments with the *ALMT1* isolines are warranted to determine how the gene expression is regulated in response to environmental stimulus and if other factors that differ between the two genetic backgrounds affect malate secretion.

Previous evidence has found that wheat and other plant species differ in the organic compounds they deposit into the soil rhizosphere (Rengel and Römheld, 2000; Sasaki et al., 2004; Zuo et al., 2014). Multiple biochemical differences were found between the bacterial communities associated with roots of different wheat lines as indicated by KO functions and pathways (**Table 4**). Zuo et al. (2014) found an increase in nitrogen-fixing and nitrifying bacteria, as well as microbial enzymes associated with nitrogen and carbon metabolism in response to different wheat lines (e.g., ‘22 Xiaoyan’). It was suggested that differences in root phytochemicals could have attributed to the observable differences. In *A. thaliana*, induction of the *AtALMT1* gene, and subsequently the increase in root exudates of MA, had the effect of recruiting bacterial species *Bacillus subtilis* (strain FB17) into the rhizosphere (Lakshmanan et al., 2013). In the present study, wheat cultivar differences in the bacterial sequence counts by KO terms could be the result of root exudates which select and increase bacteria associated with antibiotic production, sulfur, nitrogen, phosphorus, and malate responsive metabolisms (**Table 4**).

Some of the wheat cultivar-associated bacteria identified in this study may provide beneficial services to their host. Different strains of bacteria isolated from soil have been found to promote plant growth by producing plant hormones such as indole-3-acetic acid (auxin) and 1-aminocyclopropane-1-carboxylate, a precursor for ethylene (Maimaiti et al., 2007; Madhaiyan et al., 2010; Marques et al., 2010; Soltani et al., 2010). It is theorized that the increase in plant growth creates a positive feedback which increases root exudates for bacterial metabolism. Two genera in this study Amycolatopsis and Sphingomonas were found to be wheat cultivar-associated and members of these genera have been previously shown to contribute to plant health by antibiotic production (Wink et al., 2003; Chen et al., 2016) and disease suppressive effects (Vogel et al., 2012), respectively. There is further evidence to suggest that strains of *Chryseobacterium* and *Pedobacter* produce antifungal compounds against soilborne *Phytophthora* and *Rhizoctonia* species (Marques et al., 2010; Kim et al., 2012; Yin et al., 2013), although, the mechanism for this suppression is still unclear. In this study, the OTU_1302 corresponded to a *C. soldanellicola* isolate which was identified by Yin et al. (2013) and found to suppress *R. solani* in culture and in greenhouse assays. Several other suppressive bacteria were identified in that study but did not correspond to any of our wheat cultivar associated OTUs. These results indicated that disease suppressive soils, although not tested in our study, may be attributed to multiple species working in concert together (Mendes et al., 2011; Yin et al., 2013; Shen et al., 2015; Chapelle et al., 2016).

Sequencing depth played a major role in quantifying the bacterial community. Altogether, 26,604 OTUs with a ≥97% sequence similarity were found. These numbers are higher than reported in previous wheat rhizosphere studies using pyrosequencing and TRFLP (Donn et al., 2015; Corneo et al., 2016) and are consistent with other rhizosphere sequencing studies (Mendes et al., 2011; Peiffer et al., 2013; Edwards et al., 2015). Increasing sequencing depth would allow for rarer OTUs to be sequenced which may provide improved cataloging of unclassifiable or unculturable OTUs. When OTUs were filtered by a relative abundance of 0.01%, 1305 remained, suggesting a large portion of the community is composed of rarer OTUs. A recent study suggests that the roots of wild grasses such as oat (*Avena* spp.) select for rarer bacterial communities, and despite deeper 16S rDNA sequencing, these rarer bacteria were not detectable in the bulk soil (Nuccio et al., 2016). Our data is consistent with these findings and suggested a large portion of OTUs were not in high abundance but were detectable using deeper 16S rDNA sequencing.

The most dominant taxa observed in our wheat rhizospheres were in agreement with previous wheat studies but differ from those of other species. The most abundant families in the present study were Sphingobacteriaceae (Bacteroidetes; 15.5% of total sequence reads) and Gemmatimonadaceae. Previous studies complement the current study demonstrating Sphingobacteriaceae as a dominant taxon in the wheat rhizosphere (Yin et al., 2010, 2013; Wang et al., 2013; Donn et al., 2015; Corneo et al., 2016). Alternatively, the most dominant taxa for *Arabidopsis* (Bulgarelli et al., 2012; Lundberg et al., 2012) and Lettuce (Cardinale et al., 2015) was Comamonadaceae (Proteobacteria), which accounted for only 0.01% of the total sequence reads for the present study. In studies comparing wild and domesticated maize, the family Burkholderiaceae has been found to be the most dominant (Estrada et al., 2002; Szoboszlay et al., 2015). Other abundant taxa, such as Bradyrhizobiaceae and Sphingomonadaceae have also been found in the rhizospheres of wheat, *Arabidopsis*, lettuce, and sugarcane (Bulgarelli et al., 2012; Lundberg et al., 2012; Yeoh et al., 2015) and are apparently less species dependent. However, it is difficult to determine how the representative OTUs in the present study compare with other studies due to the differences in sequenced regions, platforms, and the techniques used.

We have shown wheat cultivars are involved in shaping the rhizosphere by differentially altering the bacterial community. Using deep 16S rDNA sequencing, we could characterize a larger portion of the rhizosphere community than has been previously reported. The differences in microbial communities observed on different wheat lines indicate that rhizosphere communities can be manipulated by wheat breeding. The specific community members found to be responsive to different wheat lines can be used as biomarkers for these community-altering traits. These biomarkers will assist in dissecting the genetic pathways used by these wheat hosts in recruitment, and provide the necessary tools for breeders to incorporate these favorable alleles into commercial production. A future challenge will be to determine which of these traits, and which recruited microbial community components, provide advantages to sustainable wheat production in various environments.

## Author Contributions

SH conceived the research project with input from AM and CY on technical aspects. AM and CY collected the samples, generated the amplicons and processed the sequences. AM performed the data analysis. AM and SH wrote the manuscript and all authors contributed to the manuscript revision.

## Conflict of Interest Statement

The authors declare that the research was conducted in the absence of any commercial or financial relationships that could be construed as a potential conflict of interest.
