# Supplementary material for: Community Structure, Species Variation, and Potential Functions of Rhizosphere-Associated Bacteria of Different Winter Wheat (Triticum aestivum) Cultivars
Source: Front Plant Sci. 2017 Feb 13;8:132. doi: 10.3389/fpls.2017.00132 (PMC5303725; doi:10.3389/fpls.2017.00132)
Supplement: Supplementary file 1 [file Table_1.docx]

Supplementary Table 1. Combined temperatures and precipitations for the Cook and Plant Pathology farms.

|  | 2008 | | 2009 | | 2010 | | 2012 | | 2013 | | 2014 | |
| --- | --- | --- | --- | --- | --- | --- | --- | --- | --- | --- | --- | --- |
| Month | Temp^1^ | Precip^2^ | Temp | Precip | Temp | Precip | Temp | Precip | Temp | Precip | Temp | Precip |
| Janurary | -3.2 | 63.2 | -1.4 | 70.9 | 3.0 | 13.2 | 0.4 | 73.4 | -2.2 | 29.7 | 0.7 | 48.5 |
| Feburary | 2.3 | 19.8 | 2.4 | 39.9 | 3.9 | 4.1 | 1.5 | 35.3 | 1.9 | 30.2 | -1.9 | 64.3 |
| March | 3.5 | 46.5 | 1.6 | 97.5 | 5.1 | 27.7 | 3.9 | 129.0 | 5.3 | 24.4 | 5.9 | 71.6 |
| April | 6.1 | 17.3 | 7.7 | 49.8 | 7.7 | 69.6 | 8.9 | 66.3 | 6.9 | 60.2 | 8.2 | 36.8 |
| May | 13.0 | 32.0 | 12.8 | 57.2 | 10.0 | 42.9 | 10.8 | 31.2 | 13.1 | 20.8 | 12.7 | 24.6 |
| June | 14.8 | 39.1 | 15.6 | 31.0 | 14.0 | 66.8 | 14.0 | 58.9 | 15.9 | 46.0 | 14.7 | 23.6 |
| July | 18.7 | 6.4 | 20.5 | 24.6 | 18.4 | 7.9 | 19.7 | 5.1 | 19.5 | 7.1 | 21.8 | 8.4 |
| August | 18.9 | 36.8 | 20.2 | 40.1 | 17.9 | 8.4 | 19.3 | 0.0 | 20.2 | 7.4 | 20.2 | 12.7 |
| September | 15.3 | 9.1 | 16.0 | 2.8 | 15.0 | 24.4 | 15.3 | 0.0 | 16.0 | 67.6 | 16.2 | 11.2 |
| October | 9.1 | 13.5 | 6.5 | 47.2 | 9.3 | 57.9 | 8.8 | 66.0 | 7.2 | 3.8 | 12.2 | 28.2 |
| November | 5.8 | 85.9 | 3.5 | 39.4 | 1.2 | 48.8 | 5.0 | 69.9 | 2.6 | 58.2 | 2.2 | 68.3 |
| December | -2.7 | 65.5 | -2.7 | 20.6 | 0.0 | 60.7 | 0.3 | 55.6 | -2.9 | 22.9 | 1.9 | 77.5 |

^1^ Temp is abbreviated for temperature in degrees Celsius, and is the monthly average for the given year. ^2^ Precip is abbreviated for precipitation in millimeters, and is the monthly average for the given year.
