# Supplementary material for: Community Structure, Species Variation, and Potential Functions of Rhizosphere-Associated Bacteria of Different Winter Wheat (Triticum aestivum) Cultivars
Source: Front Plant Sci. 2017 Feb 13;8:132. doi: 10.3389/fpls.2017.00132 (PMC5303725; doi:10.3389/fpls.2017.00132)
Supplement: Supplementary file 2 [file Table_2.docx]

Supplemental Table 2. Total sequence counts by cultivar for the V1-V3 regions obtained from root rhizosphere soils in the Cook and Plant Pathology farms.

|  | Cook Farm | | | Plant Pathology Farm | | |
| --- | --- | --- | --- | --- | --- | --- |
|  | Rep 1 | Rep 2 | Rep 3 | Rep 1 | Rep 2 | Rep 3 |
| Eltan | 106853 | 97290 | 93323 | 93406 | 92889 | 105998 |
| Finch | 109472 | 108640 | 112578 | 109478 | 107800 | 96163 |
| Hill81 | 142781 | 99837 | 104869 | 101681 | 95078 | 103881 |
| Lewjain | 93537 | 95476 | 104555 | 120455 | 100383 | 127057 |
| Madsen | 83503 | 107350 | 95913 | 114091 | 107916 | 103203 |
| PI561722 | 24420 | 107544 | 100992 | 106692 | 102208 | 98005 |
| PI561725 | 99655 | 92981 | 102549 | 106766 | 97022 | 94993 |
| PI561726 | 97273 | 85046 | 104499 | 126939 | 102490 | 99757 |
| PI561727 | 157334 | 101183 | 87389 | 105695 | 107350 | 105736 |
