# Supplementary material for: Community Structure, Species Variation, and Potential Functions of Rhizosphere-Associated Bacteria of Different Winter Wheat (Triticum aestivum) Cultivars
Source: Front Plant Sci. 2017 Feb 13;8:132. doi: 10.3389/fpls.2017.00132 (PMC5303725; doi:10.3389/fpls.2017.00132)

Supplemental Figure 1. An exploratory co-occurrence network showing the rhizosphere associated genera community. Nodes are labeled by genera and are colored by family level classification. The green and red lines specify significant positive and negative correlations (Spearman correlations > 0.8 or < -0.8, *P* <0.01) between two nodes, respectively.


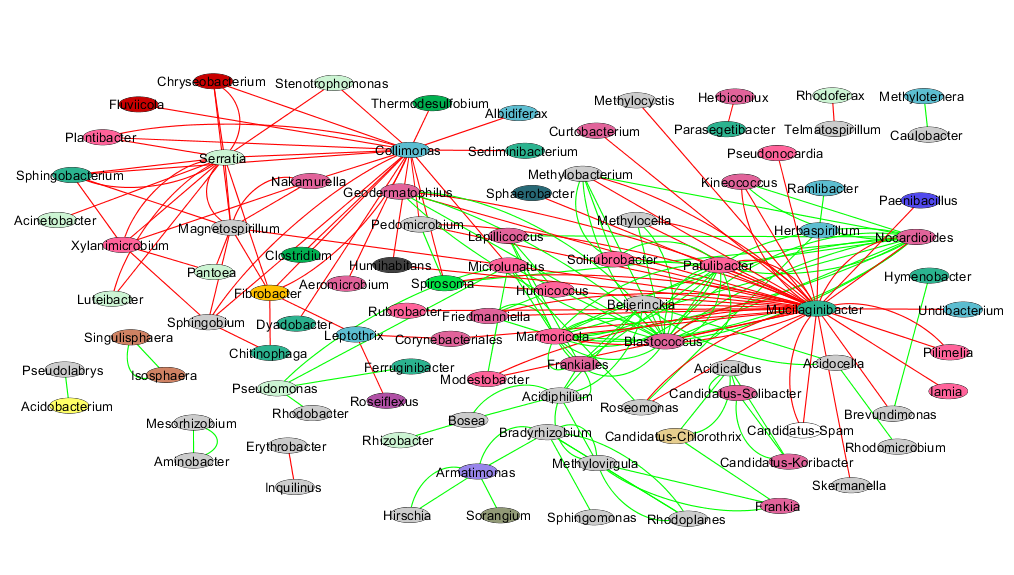

Supplement: Supplementary file 3 [file Data_Sheet_1.DOCX]
